# Response to Amir Attaran

**DOI:** 10.1371/journal.pmed.0020379

**Published:** 2005-11-29

**Authors:** John W McArthur, Jeffrey D Sachs, Guido Schmidt-Traub

**Affiliations:** **1**United Nations Millennium ProjectNew York, New YorkUnited States of America

Amir Attaran's Policy Forum [1] raises important points on the poor quality of data for some indicators used to measure progress on the Millennium Development Goals (MDGs), but, sadly, uses these findings to draw the wrong conclusions. The evidence he presents on a small number of indicators is partial, and does not justify his conclusion that the MDGs might become a liability and are doomed to fail. Quite the opposite is the case.

Of course the data on the world's extremely poor people are weak, as is just about every other aspect of efforts vis-a-vis the poor. The rich countries dramatically underinvest and make far too little effort in helping to save the poor from dying of malaria and tuberculosis (TB). It is, therefore, no surprise that developing countries and the international system lack the resources and operational support to measure malaria and TB well. Attaran's criticisms in this regard are justified and have been made by many others before him, including many professionals working for the United Nations (UN) system.

The MDGs are a political commitment made by the 189 countries represented at the 2000 Millennium Summit to halve extreme poverty in its many forms by 2015. The author ignores that such broad outcome goals adopted by world leaders are distinct from the technical question, how to define and measure corresponding indicators, which the UN has been asked to help answer. It is, therefore, inaccurate to blame the UN system for setting goals that are difficult to measure. Goal setting is the prerogative of world leaders, and they have correctly reaffirmed their commitment to the MDGs many times since 2000.

In response, the UN system has set up an active process to review indicators and data on progress toward achieving the MDGs, involving many UN organizations as well as the World Bank and the International Monetary Fund. In recent years, this interagency process has already revised several MDG indicators and issued guidance notes on how data collection can be improved. The author's assertion that the UN “shows a profound disrespect for the scientific process” [1] is wrong and misleading. The UN leadership rightly decided that the heads of state and government convening at the 2005 World Summit should focus their attention on the high-level political decisions needed to strengthen the international framework for security, development, and human rights. On the development side, the greatest priority was to cement the MDGs as operational rather than simply rhetorical targets. The world leaders did not delve into the technical issues of measurement and indicators, but this important work will continue to be addressed by UN statisticians and independent experts. Such experts have indeed been scrutinizing the definition and measurement of these indicators for some time—as did, for example, several of the UN Millennium Project task forces.

Another shortcoming of Attaran's article is that it generalizes incorrectly across the MDGs. It describes some of the toughest measurement challenges (e.g., maternal mortality and malaria), and uses them to paint all the MDGs with the same brush. In addition to the example of child mortality rates cited in the article, several other MDG indicators can be measured quite well. These indicators include anthropometric measures of malnutrition, primary school enrollment, gender parity in education, and access to basic infrastructure services.

An implication of Attaran's argument is that there should be no goals when measurement is imperfect, as it is in many countries with indicators for maternal mortality. Should world leaders, therefore, not set time-bound goals for reducing maternal mortality? This would be wrongheaded for three clear reasons. First, even with incomplete or missing data, dramatic and verifiable improvements in women's health can be achieved by investing in emergency obstetric care and other known, monitorable, and practical interventions to build and sustain primary health systems. The MDGs provide a major political and operational framework for doing this. Second, the MDGs are already promoting strengthened health systems in low-income settings, and those improved systems are key to ensuring the vital registrations that the author rightly recommends for improving the measurement of maternal mortality. Third, the very adoption of the maternal mortality goal (and others) is provoking greatly increased attention to improvements in data collection from the World Health Organization, the Gates Foundation, the World Bank, academia, and others. The MDGs should not be misunderstood as a static, one-off process.

Attaran misleads the reader when he argues that the MDGs have become “all-encompassing” catch-alls for tenuously related interventions. To say that roads and electricity are falsely linked to achieving the MDGs is incorrect, and suggests a lack of understanding of the integrated nature of development processes. Roads and electricity play a critical role in poverty reduction, access to essential health services, reduction of maternal and child mortality, and a host of other channels directly related to success in achieving the MDGs. Therefore, any strategy to achieve the MDGs needs to include these interventions. The UN Millennium Project described these linkages in the most detailed series of studies on practical approaches to achieving the MDGs that has ever been produced.

We hope that Attaran's key message on improved data collection and interpretation is heard. More and better data are certainly needed on the conditions of the world's poorest and most vulnerable people. However, even in countries with poor data systems, enough is known today to start making the practical and measurable investments in education, health, basic infrastructure, and improved environmental management that are vitally needed to cut, and eventually to end, extreme poverty. Crucially, the MDGs provide the unique framework for prompting the international cooperation that is indispensable to helping poor countries escape the poverty trap, and to benchmarking progress en route. No discussion about indicators and measurement—no matter how justified it is—should deflect from the overarching global commitment to the poorest of the poor that world leaders struck at the Millennium Summit in 2000 and reconfirmed at the World Summit in 2005.
